# Comparisons of different exercise interventions on glycemic control and insulin resistance in prediabetes: a network meta-analysis

**DOI:** 10.1186/s12902-021-00846-y

**Published:** 2021-09-06

**Authors:** Li Huang, Yingjie Fang, Lijun Tang

**Affiliations:** 1 Institute of Physical Education, Soochow University, Suzhou, 215100 Jiangsu China; 2 Shanghai Kangjian Foreign Language Experimental Middle School, Xuhui District, Shanghai, 200233 China; 3 Institute of Physical Education, Shanghai Normal University, No. 5, Lane 14, Guilin West Street, Xuhui District, Shanghai, 200234 China

**Keywords:** Prediabetes, Aerobic exercise training, Resistance training, Insulin resistance, Glycemic control, Network meta-analysis

## Abstract

**Background:**

In order to recommend the optimal type of exercise for type 2 diabetes prevention, different exercise interventions were compared with respect to their effects on glycemic control and insulin resistance.

**Methods:**

Studies on the curative effect of aerobic exercise training (AET), resistance training (RT), or control training (CT) on prediabetes were retrieved from the PubMed, Embase, SPORTDiscus, and Cochrane Library databases. Body mass index (BMI); homeostasis model assessment of insulin resistance index (HOMA-IR); and fasting blood glucose (FBG), glycated hemoglobin (HbA1c), and insulin levels were used as outcome indicators. The Q statistic was calculated to evaluate heterogeneity within studies. A fixed- or random-effects model was used for pooling data based on nonsignificant or significant heterogeneities. A consistency test was conducted using a node-splitting analysis.

**Results:**

A total of 13 eligible studies were included. The results of the direct meta-analysis indicated that AET or RT could significantly reduce the HbA1c level in prediabetic individuals compared to CT [AET vs. CT: standardized mean difference (SMD) = − 0.6739, 95% confidence interval (CI) = − 0.9424 to − 0.4055 to RT vs. CT: SMD = − 1.0014, 95% CI = − 1.3582 to − 0.6446]. The findings from the network meta-analysis showed that there were no statistical differences among the four comparisons for all the indicators except for lower HbA1c level (SMD = − 0.75, 95% CI = − 1.31 to − 0.19) and HOMA-IR (SMD = − 1.03, 95% CI = − 1.96 to − 0.10) in the AET group than in the CT group. In addition, prediabetic individuals in the AET + RT group showed greater control of BMI and insulin and FBG levels than those in the other groups, whereas AET was the most effective in controlling HbA1c and HOMA-IR levels in prediabetic individuals.

**Conclusion:**

AET, AET + RT, and RT exerted beneficial effects on insulin resistance and glycemic control in prediabetic patients. From the existing data, AET or AET + RT is preferentially recommended for these patients, although further studies may unveil RT as a promising therapy. Benefits from all types of exercise seem to occur in an intensity-dependent manner.

**Supplementary Information:**

The online version contains supplementary material available at 10.1186/s12902-021-00846-y.

## Background

Prediabetes, also defined as impaired glucose regulation (IGR), is characterized by increased glycated hemoglobin (HbA1c) level, impaired fasting glucose (IFG), impaired glucose tolerance (IGT), or a combination of IFG and IGT. It is an intermediate state between normal glycometabolism and diabetes and presents with poor glucose regulation [1]. Based on the World Health Organization definition, a person with a high fasting blood glucose (FBG) level (6.1–6.9 mmol/L) in the morning after an overnight fast is considered to have IFG [2]. Individuals with IGT have an increased postprandial blood glucose level [3]. Insulin resistance and pancreatic β-cell dysfunction are considered to be the two main causes for the development of IFG and IGT [4, 5].

Reportedly, individuals with prediabetes have a 30–70% chance of developing type 2 diabetes over the next 4–30 years [6]. In China, the overall prevalence of diabetes is estimated to be 10.9%, whereas that of prediabetes is estimated to be 35.7% [7]. In addition, prediabetes and type 2 diabetes can cause cardiovascular complications, which may contribute to an elevated risk of mortality [8]. With the increasing prevalence of prediabetes and type 2 diabetes in China, the prevention of prediabetes may be an important strategy for delaying the onset of type 2 diabetes and its associated complications.

Several factors such as smoking, harmful drinking, obesity, and abnormal cholesterol and triglyceride levels may lead to an increased risk of prediabetes [9]. It has been suggested that lifestyle interventions involving increased physical activity and dietary changes to promote low energy intake may prevent type 2 diabetes [10]. Interestingly, exercise-induced weight reduction is superior to dieting for improving insulin resistance in individuals with obesity [11]. The underlying mechanism may be that exercise-induced weight reduction can suppress unnecessary gluconeogenesis via the activation of mitochondrial oxidative capacity and decreased endogenous glucose production [12].

Although several exercise interventions including aerobic exercise training (AET), resistance training (RT), or a combination of AET + RT have been used for the prevention of diabetes in prediabetic individuals [13–15], a comprehensive comparison of these interventions in this group of people has not been described. Therefore, to investigate the optimal type of exercise intervention for prediabetic individuals, we conducted both direct and network meta-analyses to evaluate the effects of these different types of exercise intervention on five indicators: body mass index (BMI); homeostasis model assessment of insulin resistance index (HOMA-IR); and FBG, HbA1c, and insulin levels.

## Methods

### Search strategy

Studies on the curative effect of AET or RT in prediabetes were searched for in the PubMed, Embase, SPORTDiscus, and Cochrane Library databases up to September 11, 2020. The search terms used were (pre-diabetes OR prediabetic OR “impaired glucose regulation” OR IGR OR “impaired fasting glucose” OR IFG OR “impaired glucose tolerance” OR IGT OR “glucose metabolism disorders” OR “glucose alterations” OR “hyperglycemia” OR “dysglycemia”) AND (“exercise” OR “weightlifting” OR “aerobic exercise” OR “aerobic training” OR “aerobic therapy” OR “movement” OR “physical therapy” OR “resistance exercise” OR “physical activity” OR “resistance training” OR “resistance”). Subject and free words were combined in order to search for related articles, and the search format was adjusted according to the characteristics of the database (the specific retrieval steps of each database are shown in Supplementary Tables 1, 2, 3 and 4).

### Inclusion and exclusion criteria

Studies were required to meet the following inclusion criteria: 1) the curative effects of any two exercise interventions [AET, RT, AET + RT, or control training (CT)] on prediabetes were evaluated; AET consisted of specifically designed and supervised exercise sessions that were rhythmic, dynamic, and aerobic in nature, including walking, running, dancing, skating, swimming, cross-country skiing, and engaging in endurance activities [16]; RT focused on strength and power exercises for the lower extremities, trunk, and upper extremities performed using regular resistance equipment [17]; AET + RT was a combination of aerobic and resistance exercise programs; and CT entailed an explanation of the health benefits of exercise, but no supervised exercise was recommended; 2) at least one of the main outcomes was reported (BMI, FBG levels, or any change in the values of HbA1c, insulin, or HOMA-IR), and 3) the study design was a randomized controlled study (RCT).

Studies were excluded if they met any one of the following criteria: 1) the data provided were incomplete and could not be used in the statistical analysis; 2) the article was a review, comment, or letter; and 3) the study was repeatedly published or the same population was used for multiple studies, in which case only the most recent study or the study with the most information was included.

### Data extraction and quality evaluation

The following information was independently extracted by two investigators: the study characteristics (first author of study, study region, publication year, follow-up time, type of prediabetes, type of exercise, and total number of participants) and the characteristics of the participants (age, sex ratio, and BMI).

In addition, the quality of the studies was also evaluated by two investigators using the Cochrane Collaboration’s risk-of-bias tool [18], which assesses selection, performance, detection, attrition, and reporting biases.

Disputes regarding the extraction of data and assessment of the quality of the studies between the two investigators were resolved through consultation and discussion with a third investigator.

### Statistical analysis

Both direct and network (or indirect) meta-analyses were performed on the pooled data. The “meta” package in R 3.4.3 software was used to merge the data for the direct meta-analyses. The effect size for variables was indicated as standardized mean difference (SMD) and its 95% confidence interval (CI). I^2^ statistics were calculated in order to assess the level of heterogeneity among the studies. Where there were statistical differences in the heterogeneity test statistics (I^2^ > 50%), a random-effects model was applied to calculate the pooled value; otherwise, a fixed-effects model was used [19].

For the network meta-analysis, the “netmeta” package in the R 3.4.3 software was used. A Cochran’s Q statistic was calculated to evaluate heterogeneity among the studies. For evaluating the pooled data with a *P* value of the Q statistic > 0.05, a fixed-effects model was chosen; otherwise, a random-effects model was used [20]. In the network meta-analysis, the ranking of all the interventions was based on P-scores; the higher the P-score of the intervention, the better the curative effect compared to others [21].

### Sensitivity analysis and consistency tests

Both fixed- and random-effects models were used to perform sensitivity analyses of the P-score. A consistency test was conducted using node-splitting analysis, and the *P*-values of the node-splitting analysis were used to compare the results between the direct and indirect analyses. If there were no significant differences between the results of the direct and indirect analyses (*P* > 0.05), a consistency model was applied to pool the data; otherwise, an inconsistency model was adopted.

## Results

### Eligible studies

**Fig. 1:**
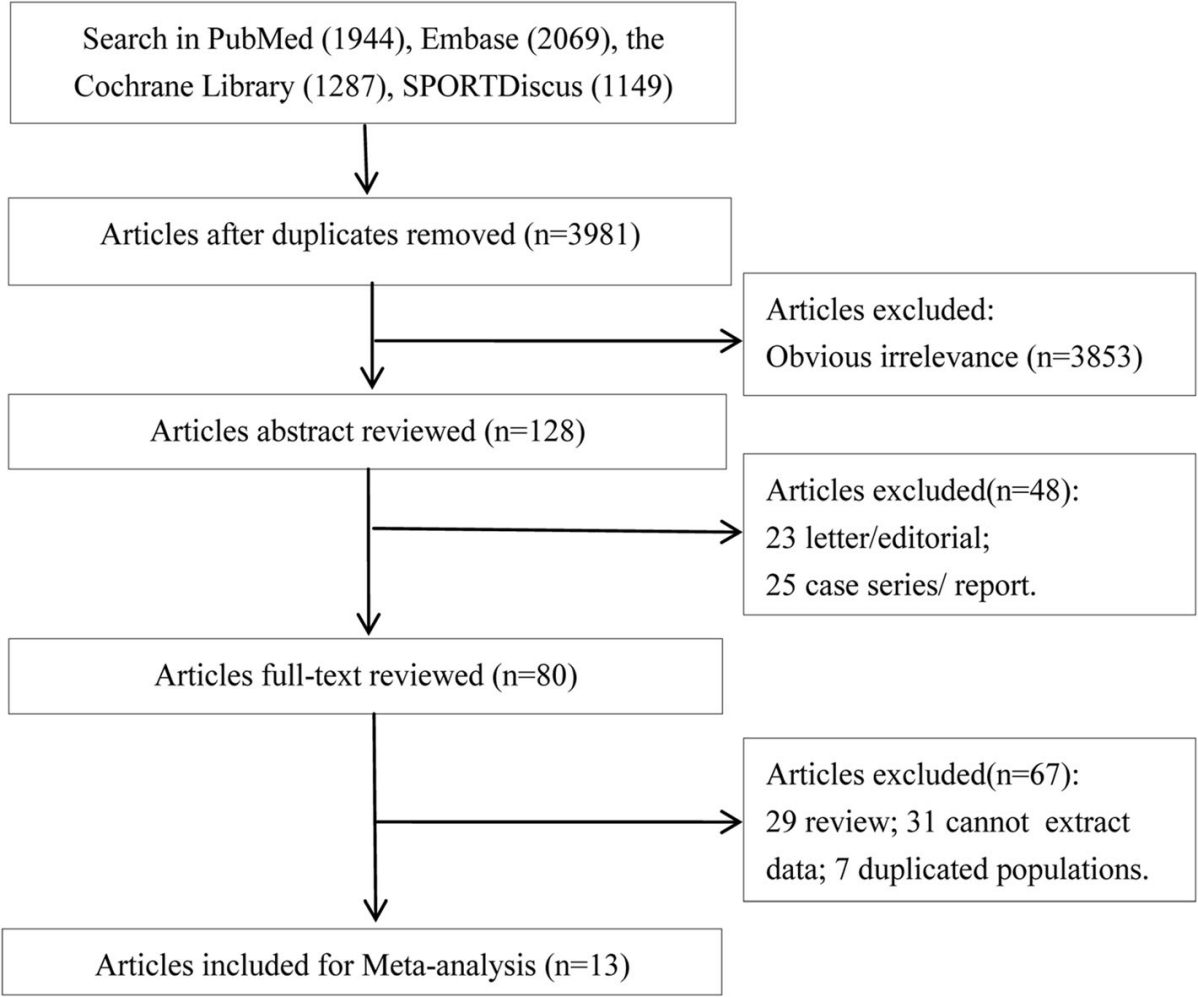
Literature search and study selection process

The study screening process is presented in Fig. 1. In total, 6449 relevant articles were retrieved from the PubMed (1944), Embase (2069), SPORTDiscus (1149), and Cochrane Library (1287) databases based on the preliminary search strategy. After removing 2468 duplicates, 3981 articles were analyzed further. Of these articles, 3853 were excluded for being irrelevant based on a review of their titles and abstracts. Using the full-text reviewing process in the “netmeta” package, the remaining 128 articles were further filtered, and 115 articles that did not meet the criteria were excluded. The excluded articles included 25 case series/reports, 23 letters/comments, 29 reviews/meta-analyses, 7 studies with duplicated populations, and 31 articles without available data. As result, a total of 13 eligible studies were used in the meta-analysis [13–17, 22–29].

### Characteristics of eligible studies

The characteristics of the 13 eligible studies are presented in Table 1. These studies were published between 1998 and 2019. The studies were conducted in China, Chile, Austria, Belgium, Netherlands, United States, Canada, Germany, Finland, and Sweden. AET, CT, RT, and AET + RT were the main types of exercise interventions implemented. In total, 567 participants were recruited, of whom 158 underwent AET, 249 CT, 89 RT, and 71 AET + RT. Based on BMI values, the majority of the participants were obese, and most of the studies had a 12-week follow-up. The prediabetic subjects in the included studies comprised mainly individuals with IFG and IGT. In most of the studies, the baseline characteristics of age, pre-training BMI, and sex of the subjects among different groups of the included studies were not significantly different, except for significant differences in age at baseline among groups in the study by Roumen et al. (2008) and in pre-training BMI at baseline among the groups in the study by Venojärvi et al. (2013).

**Table 1:** Characteristics of the 13 included studies

Study	Location	Duration of intervention	Type	Group	n	Sex (M/F)	Age (years)	BMI (kg/m^2^)	Intervention program
Alvarez et al., 2012	Chile	12 weeks	Prediabetes	RT	8	0/8	NA	NA	Performed using regular resistance equipment.
				CT	13	0/13	NA	NA	As usual.
Burtscher et al., 2009	Austria	12 months	IFG	AET	18	16/20	57.5 ± 6.9	30.6 ± 4.1	Supervised, progressive, individually tailored aerobic exercises for 1 h, twice a week.
				CT	18			27.3 ± 3.8	As usual.
Desch et al., 2010	Germany	6 months	Prediabetes	AET	14	11/3	62.3 ± 6.2	29.8 ± 4.0	Daily home-based sessions on a stationary bicycle at 75% of maximum heart rate and additional supervised indoor and outdoor group exercise sessions twice a week.
				CT	12	8/4	62.3 ± 6.5	31.3 ± 3.9	As usual.
Eriksson et al., 1998	Finland	10 weeks	IGT	AET	7	3/4	60 ± 5	NA	Supervised group exercise sessions (aerobic endurance training 1 h/week, target heart rate approximately 60% of maximum)
				CT	7	4/3		NA	Informed about the positive effects of exercise but no individual exercise programs.
Fritz et al., 2012	Sweden	NA	IGT	AET	14	5/9	59.1 ± 6.2	32.0 ± 5.2	Instructed to increase their weekly level of physical activity by 5 h of walking.
				CT	21	10/11	61.8 ± 3.4	30.8 ± 3.5	As usual.
Malin et al., 2012	USA	12 weeks	Prediabetes	AET + RT	8	3/5	45.4 ± 8.0	33.5 ± 4.1	Subjects performed aerobic and resistance exercise on the 1st and 3rd day of each week. To minimize muscle soreness, only aerobic training was performed on the 2nd day.
				CT	8	2/6	49.8 ± 10.9	34.0 ± 6.3	As usual.
Marcell et al., 2005	USA	16 weeks	Insulin resistance	AET	20	NA	44.4 ± 6.5	32.5 ± 5.3	30 min of activity 5 days/week taking into account preferred activities (mostly walking or jogging outdoors or on a treadmill).
				CT	14	NA	44.1 ± 9.5	35.3 ± 3.7	As usual.
Marcus et al., 2005	USA	12 weeks	IGT	RT	10	0/10	56.3 ± 6.4	28.5 ± 3.7	High-force lower extremity extensor muscle contractions for 3 nonconsecutive days/week.
				CT	6	0/6	53.2 ± 6.5	32.2 ± 4.0	No participation in a supervised exercise program.
Roumen et al., 2008	Netherlands	3 years	IGT	AET + RT	52	28/24	54.2 ± 5.8	29.6 ± 3.8	Physical activity at least 30 min a day for at least 5 days a week, at an intensity of at least 70% of their VO_2_max.
				CT	54	30/24	58.4 ± 6.8*	29.2 ± 3.3	Briefly informed about the beneficial effects of a healthy diet and physical activity, but no individual advice provided.
Rowan et al., 2016	Canada	12 weeks	Prediabetes	AET	11	3/8	53.6 ± 8.21	32.0 ± 4.6	Supervised exercise (high-intensity interval or intermittent training) in the laboratory 3 times/week for 36 sessions.
				CT	10	3/7	47.7 ± 6.92	30.8 ± 8.5	As usual.
Venojarvi et al., 2013	Finland	12 weeks	IGT	CT	40	40/0	54 ± 7.2	28.6 ± 3.0	Informed about the health benefits of exercise, but no supervised exercise provided.
				AET	39	39/0	55 ± 6.2	30.0 ± 3.4	Aerobic exercise sessions carried out at strain levels increasing from 55 to 75% of heart rate reserve (weeks 1–4 at 55%, weeks 5–8 at 65%, and weeks 9–12 at 75%).
				RT	36	36/0	54 ± 6.1	30.3 ± 3.2*	Performed using regular resistance equipment with training focus on strength and power exercises of the lower extremities and trunk, but also muscles of the upper extremities were trained.
Wens et al., 2017	Belgium	12 weeks	IGT	CT	11	2/9	48 ± 9	27.1 ± 4.4	As usual.
				AET + RT	11	5/6	47 ± 9	24.4 ± 3.8	Cycling and treadmill walking/running, moderate to high intensity resistance training (leg press, leg curl, leg extension, vertical traction, arm curl, and chest press).
Yan and Dai, 2019	China	12 months	Prediabetes	AET	35	10/25	64.23 ± 5.75	22.4 (21.4, 26.0)	Required participants to exercise 3 days/week for 60 min/session at 60 to 70% of their maximum heart rate.
				RT	35	15/20	62.06 ± 8.11	25.1 (22.7, 26.7)	Resistance training 3 days/week with a bungee cord, 50 min/session.
				CT	35	15/20	60.31 ± 7.56	24.6 (21.7, 27.6)	Maintain usual habits and received no structured exercise intervention.

*Abbreviations*: *AET* aerobic exercise training, *BMI* body mass index, *CT* control training, *F* female, *IFG* impaired fasting glucose, *IGT* impaired glucose tolerance, *M* male, *NA* not available, *RT* resistance training, *VO*_*2*_*max* maximal oxygen consumption

*P < 0.05

### Quality evaluation

**Fig. 2:**
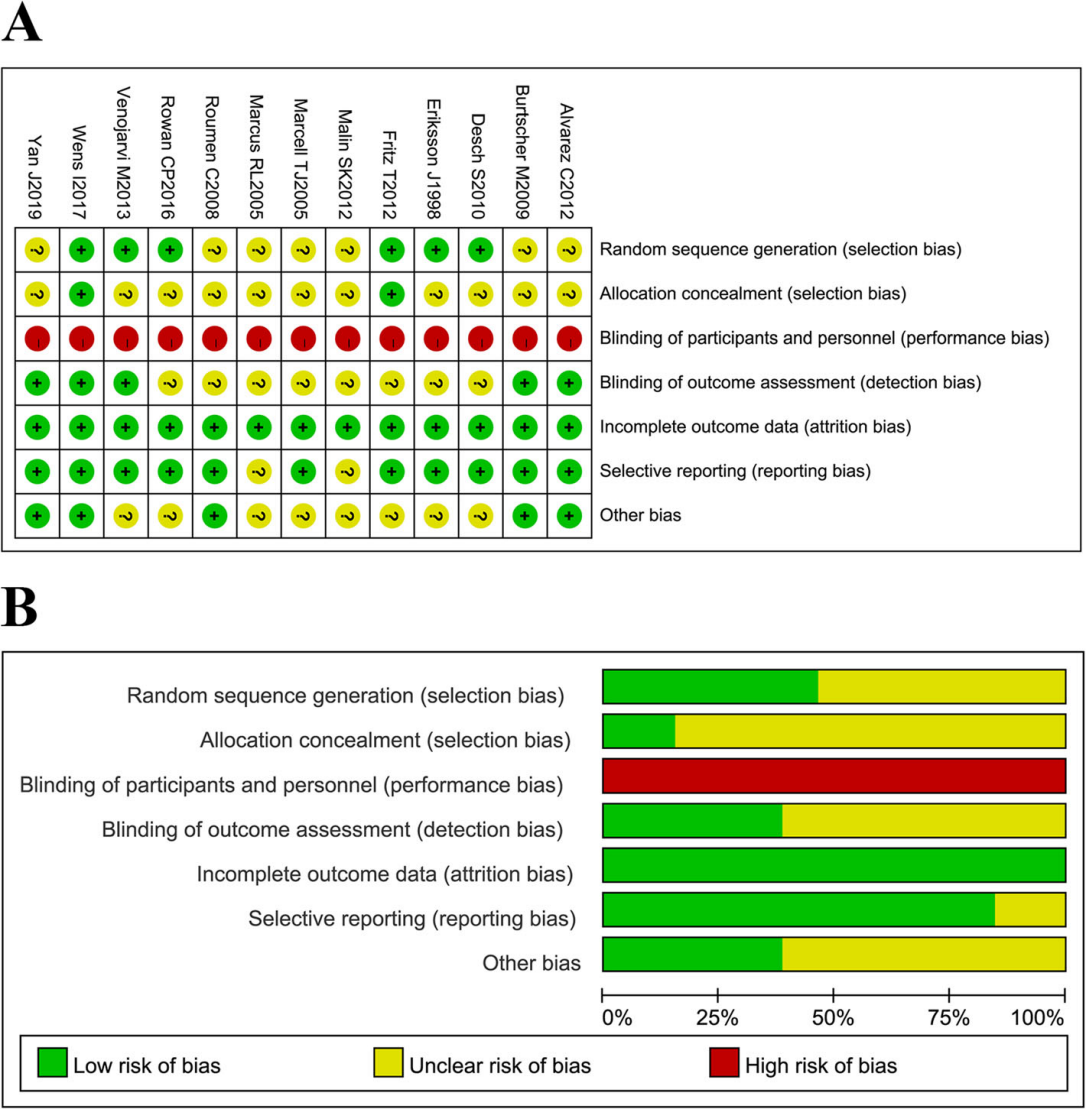
Quality assessment of the included studies. (a) Risk of bias for each included study. (b) Summary of bias risk. “+” represents low risk of bias; “–” represents high risk of bias; and “?” represents unclear risk of bias

The quality evaluation showed that all the included studies demonstrated a high risk of performance bias and that most of the studies presented an unclear risk of detection, allocation concealment, and any other biases. However, most of the studies had a low risk of random sequence generation, attrition, and reporting biases. Overall, the quality of the included studies was moderate (Fig. 2).

### Direct meta-analysis results

A direct meta-analysis was conducted to compare the efficacy of different exercise interventions in prediabetic patients.

A fixed-effects model was used to pool data with nonsignificant heterogeneity (*P* > 0.05) of the comparisons involving AET vs. CT and RT vs. CT for BMI and AET vs. CT and RT vs. AET for HbA1c levels, whereas a random-effects model was used to pool data of the comparisons involving AET vs. CT, AET + RT vs. CT, RT vs. AET, and RT vs. CT for FBG levels; RT vs. CT for HbA1c levels; AET vs. CT and RT vs. CT for insulin levels; and AET vs. CT, RT vs. AET, and RT vs. CT for HOMA-IR, all of which had significant heterogeneity (*P* < 0.05).

The pooled results showed that HbA1c levels in prediabetic individuals after AET or RT interventions were significantly reduced compared to that in individuals after CT interventions (AET vs. CT, SMD = − 0.6739, 95% CI = − 0.9424 to − 0.4055; RT vs. CT, SMD = − 1.0014, 95% CI = − 1.3582 to − 0.6446) (Table 2 and Fig. S1). However, there were no statistically significant differences for other comparisons of each indicator (Table 2 and Figs. S2, S3, S4 and S5). Notably, the results of comparisons from fewer than two included studies were not considered in our analysis because there was no statistical power for results from only one study and the reliability and validity of the findings were limited.

**Table 2:** Results of direct meta-analysis

Variable	Group	k	SMD (95% CI)	Q	I^2^ (%)	*P-*value	Model
BMI	AET vs. CT	5	−0.1500 [− 0.4464 to 0.1465]	7.18	44.3	0.127	Fixed
	AET + RT vs. CT	1	−0.2653 [− 0.6478 to 0.1172]	0.00	–	–	–
	RT vs. CT	2	−0.2336 [− 0.6490 to 0.1818]	0.23	0.0	0.634	Fixed
	AET vs. RT	1	0.2708 (− 0.2000 to 0.7416)	0.00	–	–	–
FBG	AET vs. CT	7	0.3310 [−0.4398 to 1.1018]	53.73	88.8	< 0.001	Random
	AET + RT vs. CT	2	−0.7787 [−1.9115 to 0.3540]	5.10	80.4	0.02	Random
	RT vs. AET	2	−0.4262 [− 1.3347 to 0.4824]	7.40	86.5	0.007	Random
	RT vs. CT	4	0.0057 [− 0.9556 to 0.9671]	25.48	88.2	< 0.001	Random
HbA1c	AET vs. CT	5	−0.6739 [− 0.9424 to − 0.4055]	6.17	35.2	0.187	Fixed
	AET + RT vs. CT	1	0.0245 [− 0.3563 to 0.4053]	0.00	–	–	–
	RT vs. AET	2	0.1705 [− 0.1564 to 0.4975]	1.14	12.0	0.288	Fixed
	RT vs. CT	2	−1.0014 [− 1.3582 to − 0.6446]	17.50	94.3	< 0.001	Random
Insulin	AET vs. CT	5	−0.1425 [−2.0375 to 1.7525]	100.54	96.0	< 0.001	Random
	AET + RT vs. CT	1	−0.8934 [−1.9362 to 0.1494]	0.00	–	–	–
	RT vs. AET	1	0.8907 [0.4146 to 1.3668]	0.00	–	–	–
	RT vs. CT	3	−0.9305 [−2.0838 to 0.2228]	12.11	83.5	< 0.001	Random
HOMA-IR	AET vs. CT	4	−1.0353 [−2.2784 to 0.2078]	46.72	93.6	< 0.001	Random
	AET + RT vs. CT	1	−0.5054 [−0.8925 to − 0.1183]	0.00	–	–	–
	RT vs. AET	2	0.2969 [−0.4154 to 1.0091]	4.65	78.5	0.031	Random
	RT vs. CT	3	−0.9836 [−2.2509 to 0.2837]	25.30	92.1	< 0.001	Random

*Abbreviations*: *AET* aerobic exercise training, *BMI* body mass index, *CI* confidence interval, *CT* control training, *FBG* fasting blood glucose, *HbA1c* glycated hemoglobin, *HOMA-IR* homeostasis model assessment of insulin resistance index, *RT* resistance training, *SMD* standardized mean difference

CIs crossing the zero line represent no statistically significant difference between any interventions, and CIs that are either greater or lesser than 0 represent statistically significant differences. *P* < 0.05 indicates significant heterogeneity among included studies

### Network meta-analysis results

**Fig. 3:**
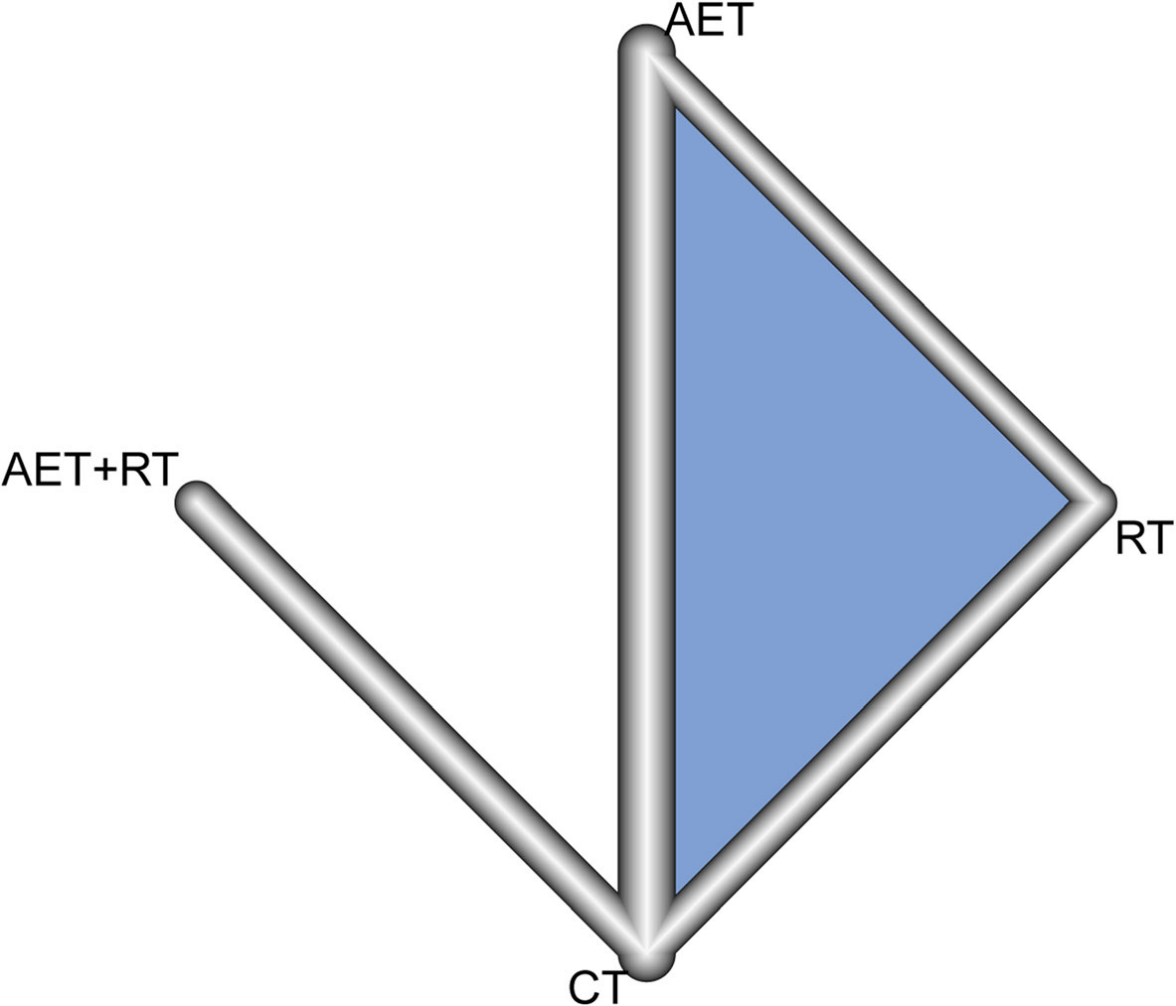
Network construction diagram. The lines between any two exercise interventions represent the comparisons of these two interventions that were reported. The thickness of the line is proportional to the number of studies that have compared these two interventions

As only pairwise comparisons of exercise interventions could be obtained by the direct meta-analysis, a network meta-analysis was performed to compare the efficacies of the four exercise interventions. The network construction diagram showed that only four exercise intervention comparisons (RT vs. CT, RT vs. AET, AET + RT vs. CT, and AET vs. CT) were reported in the included studies, and most of the included studies compared the efficacy between AET and CT (Fig. 3). Based on the Q statistic value, a random-effects model was used for the network meta-analysis. The findings of the network meta-analysis showed that the HbA1c level (SMD = − 0.75, 95% CI = − 1.31 to − 0.19) and HOMA-IR (SMD = − 1.03, 95% CI = − 1.96 to − 0.10) for prediabetic individuals after an AET intervention were significantly lower than those after a CT intervention. Although there was no statistically significant difference among the other comparisons for all indicators (BMI; HOMA-IR; and FBG, HbA1c, insulin levels), greater decreases in BMI (P-score = 0.7564) and FBG (P-score = 0.8351) and insulin (P-score = 0.6462) levels were seen in the AET + RT group than in other groups. In addition, AET was more effective for the control of HbA1c level and HOMA-IR in prediabetic individuals compared to other interventions (Tables 3 and 4).

**Table 3:** The results of network meta-analysis

	AET			
BMI	0.24 [− 0.24 to 0.72]	AET + RT		
	0.02 [− 0.31 to 0.27]	− 0.27 [− 0.65 to 0.12]	CT	
	0.23 [− 0.18 to 0.64]	− 0.01 [− 0.56 to 0.53]	0.25 [− 0.14 to 0.64]	RT
	AET			
FBG	0.88 [−0.42 to 2.19]	AET + RT		
	0.31 [− 0.31 to 0.93]	−0.58 [−1.73 to 0.58]	CT	
	0.38 [−0.50 to 1.25]	− 0.51 [− 1.90 to 0.89]	0.07 [− 0.72 to 0.86]	RT
	AET			
HbA1c	−0.77 [−2.04 to 0.49]	AET + RT		
	−0.75 [−1.31 to-0.19]	0.02 [−1.11 to 1.16]	CT	
	−0.32 [−1.09 to 0.44]	0.45 [− 0.91 to 1.82]	0.43 [− 0.34 to 1.19]	RT
	AET			
Insulin	0.76 [−3.01 to 4.54]	AET + RT		
	−0.13 [−1.63 to 1.37]	− 0.89 [−4.36 to 2.57]	CT	
	0.30 [−1.91 to 2.51]	−0.46 [−4.41 to 3.48]	0.43 [− 1.46 to 2.32]	RT
	AET			
HOMA-IR	−0.52 [−2.57 to 1.52]	AET + RT		
	−1.03 [− 1.96 to-0.10]	−0.51 [− 2.33 to 1.32]	CT	
	−0.15 [− 1.29 to 0.98]	0.37 [−1.73 to 2.47]	0.87 [− 0.17 to 1.92]	RT

*Abbreviations*: *AET* aerobic exercise training, *BMI* body mass index, *CT* control training, *FBG* fasting blood glucose, *HbA1c* glycated hemoglobin, *HOMA-IR* homeostasis model assessment-insulin resistance index, *RT* resistance training

**Table 4:** P-score distributions for each indicator

BMI			FBG			HbA1c			Insulin			HOMA-IR		
Variable	Fixed	Random	Variable	Fixed	Random	Variable	Fixed	Random	Variable	Fixed	Random	Variable	Fixed	Random
AET	0.7564	0.7564	RT	0.9769	0.8351	RT	0.9969	0.8916	AET	0.7907	0.6462	AET	0.9263	0.7602
AET + RT	0.7505	0.7505	AET	0.5237	0.5358	AET	0.6619	0.6036	CT	0.5391	0.5624	CT	0.6554	0.6601
CT	0.2830	0.2830	CT	0.4901	0.4774	AET + RT	0.1573	0.2855	RT	0.6537	0.4360	RT	0.4166	0.4601
RT	0.2100	0.2100	AET + RT	0.0093	0.1516	CT	0.1838	0.2193	AET + RT	0.0164	0.3554	AET + RT	0.0017	0.1196

*Abbreviations*: *AET* aerobic exercise training, *BMI* body mass index, *CT* control training, *FBG* fasting blood glucose, *HbA1c* glycated hemoglobin, *HOMA-IR* homeostasis model assessment-insulin resistance index, *RT* resistance training

### Sensitivity analysis and consistency tests

In order to evaluate the stability of the network meta-analysis results, both fixed- and random-effects models were used to pool the data. Notably, the results showed a consistency in the *P*-values for each indicator in both models, indicating the stability of the network meta-analysis results (Table 4). In addition, the node-splitting analysis demonstrated that the results from both direct and indirect analyses were consistent (*P* > 0.05 for all indicators) (Table 5).

**Table 5:** Results of the consistency test

Variable	Comparison	Studies providing direct evidence		Estimated treatment effect (SMD)				Test for disagreement (direct vs. indirect)	
		Number	Proportion	NMA (95% CI)	Direct (95% CI)	Indirect (95% CI)	Difference (95% CI)	Z-value	*P*-value
BMI	AET:CT	5	0.98	− 0.02 [− 0.31 to 0.27]	− 0.03 [− 0.33 to 0.26]	− 0.49 [− 1.45 to 2.42]	− 0.52 [− 2.48 to 1.44]	− 0.52	0.6042
	AET:RT	1	0.75	0.23 [−0.18 to 0.64]	0.27 [− 0.20 to 0.74]	0.11 [− 0.70 to 0.93]	0.16 [− 0.79 to 1.10]	0.33	0.7435
	CT:RT	2	0.88	0.25 [−0.14 to 0.64]	0.25 [−0.17 to 0.66]	0.31 [− 0.81 to 1.42]	−0.06 [− 1.25 to 1.13]	−0.10	0.9241
FBG	AET:CT	7	0.96	0.31 [−0.31 to 0.93]	0.33 [−0.30 to 0.96]	− 0.15 [− 3.31 to 3.00]	0.48 [− 2.74 to 3.70]	0.29	0.7698
	AET:RT	2	0.62	0.38 [−0.50 to 1.25]	0.43 [−0.68 to 1.54]	0.29 [−1.12 to 1.70]	0.14 [− 1.66 to 1.93]	0.15	0.8806
	CT:RT	4	0.89	0.07 [−0.72 to 0.86]	0.01 [−0.82 to 0.84]	0.58 [−1.85 to 3.00]	− 0.57 [−3.13 to 1.99]	−0.43	0.6638
HbA1c	AET:RT	2	0.86	−0.32 [−1.09 to 0.44]	− 0.58 [− 1.40 to 0.25]	1.25 [− 0.80 to 3.30]	−1.83 [−4.03 to 0.38]	−1.62	0.1047
	CT:RT	2	0.86	0.43 [−0.34 to 1.19]	0.68 [− 0.14 to 1.51]	−1.14 [− 3.18 to 0.90]	1.82 [− 0.38 to 4.03]	1.62	0.1046
Insulin	AET:CT	5	0.96	−0.13 [−1.63 to 1.37]	− 0.14 [− 1.67 to 1.39]	0.17 [−7.34 to 7.68]	−0.31 [−7.97 to 7.35]	−0.08	0.9362
	AET:RT	1	0.44	0.30 [−1.91 to 2.51]	−0.92 [−4.26 to 2.41]	1.25 [− 1.69 to 4.19]	−2.17 [−6.62 to 2.28]	−0.96	0.3385
	CT:RT	3	0.92	0.43 [−1.46 to 2.32]	0.88 [−1.09 to 2.85]	−4.68 [− 11.32 to 1.98]	5.55 [− 1.37 to 12.48]	1.57	0.1162
HOMA-IR	AET:CT	4	0.96	−1.03 [− 1.96 to −0.10]	−1.03 [− 1.98 to − 0.08]	−0.95 [−5.50 to 3.59]	−0.08 [− 4.72 to 4.56]	−0.03	0.9738
	AET:RT	2	0.76	−0.15 [− 1.29 to 0.98]	− 0.29 [− 1.59 to 1.01]	0.30 [−2.04 to 2.64]	−0.59 [−3.27 to 2.08]	−0.43	0.6647
	CT:RT	3	0.91	0.87 [−0.17 to 1.92]	0.98 [−0.12 to 2.08]	0.20 [−3.67 to 3.28]	1.18 [−2.46 to 4.82]	0.63	0.5261

*Abbreviations*: *AET* aerobic exercise training, *BMI* body mass index, *CI* confidence interval, *CT* control training, *FBG* fasting blood glucose, *HbA1c* glycated hemoglobin, *HOMA-IR* homeostasis model assessment-insulin resistance index, *NMA* network meta-analysis, *RT* resistance training, *SMD* standardized mean difference

## Discussion

This investigation included 13 studies to evaluate the effect of RT, AET, AET + RT, and CT interventions on five indicators (BMI; HOMA-IR; and FBG, HbA1c, and insulin levels) to evaluate prediabetes risk reduction using direct and network meta-analyses. The results of the direct meta-analysis indicated that AET or RT interventions caused a significantly greater reduction in HbA1c levels in prediabetic individuals than that caused by CT intervention. The results of the network meta-analysis revealed that HbA1c levels and HOMA-IR in prediabetic individuals after AET intervention were significantly lower than those after CT intervention. Although no significant differences in all indicators among the AET, AET + RT, and RT groups were observed in the direct and network meta-analyses, prediabetic individuals in the AET group experienced better curative effects with respect to HbA1c level and HOMA-IR compared to the other groups; the highest curative effect on BMI and FBG and insulin levels was observed with AET + RT intervention. Taken together, AET, AET + RT, and RT interventions exerted beneficial effects on prediabetic patients; however, AET or AET + RT interventions were preferentially recommended for these patients.

HbA1c level as a glycemic control indicator and HOMA-IR as an insulin resistance indicator are commonly used for evaluating the effects of diabetes. Different exercise interventions have different beneficial effects on these indicators. It has been suggested that AET intervention results in a greater reduction of HbA1c level than RT intervention in patients with type 2 diabetes [30]. In addition, RT or AET intervention alone can significantly decrease HbA1c levels in patients with type 2 diabetes to a greater extent than CT intervention [31]. This was further confirmed by the results obtained from the direct meta-analysis. In addition, HOMA-IR values in prediabetic subjects have been shown to be significantly reduced following exercise compared to those in a control group, and insulin secretion adjusts in an exercise intensity-dependent manner relative to the level of insulin resistance [32].

Moreover, AET intervention improves insulin sensitivity in adolescents with obesity and low HOMA-IR [33]. Furthermore, the insulin level and HOMA-IR were significantly lower in prediabetic patients with coronary artery disease after AET intervention than in the control group [22]. These findings are consistent with the results of this study’s network meta-analysis, which showed significantly reduced HOMA-IR in the AET group compared to that in the CT group. In addition, although there were no significant differences in benefits with respect to HbA1c level and HOMA-IR among the three exercise groups (AET, AET + RT, and RT), a greater improvement of HbA1c level and HOMA-IR was seen in prediabetic individuals in the AET group than in the other groups. Similarly, small but statistically nonsignificant decreases in insulin level and HOMA-IR were found in an AET group compared to that of an RT group [34].

Reportedly, glucose uptake and utilization are increased with AET intervention via activation of AMP-activated protein kinase, whereas RT intervention can enhance glucose uptake and reduce blood glucose by resistance exercise-induced glucose transporter 4 translocation [35]. The effect of RT and AET interventions on glucose uptake regulation have different mechanisms. Consequently, peak oxygen consumption is increased more in the AET + RT and AET groups than in the RT group, whereas strength is increased more in the AET + RT and RT groups than in the AET group [36]. Luo et al. showed that both RT and AET interventions can remarkably reduce FBG levels in prediabetic participants compared with CT interventions, but no significant difference was found between the RT and AET groups [37]. Liu et al. also demonstrated that there were no marked changes in BMI and fasting insulin levels between the walking+exercise and walking+resistance exercise interventions groups [38]. Similarly, in the present study, although there were no significant differences in FBG or insulin levels and BMI in prediabetic individuals after the four exercise interventions, AET + RT was shown to be more effective in improving BMI and FBG and insulin levels than the other interventions. It has been reported that enhanced glucose disposal is related to increased muscle density [39]. The AET + RT group showed a significantly greater increase in muscle density than the AET only group [40]. This partially explains this study’s results indicating that the AET + RT and RT groups showed a slightly greater benefit in terms of FBG levels and BMI than the AET group.

Although previous meta-analyses have compared the effectiveness of different exercise interventions in diabetic and prediabetic patients [40–42], their findings differed substantially from those of the present study. A meta-analysis of seven studies by De Nardi et al. [40], with study subjects and intervention groups that differed from those of the present study, showed that high-intensity interval training significantly promotes maximal oxygen consumption (VO_2_max). However, in patients with prediabetes and type 2 diabetes, there were no differences in the cardiometabolic markers including HbA1c levels, high-density lipoprotein and low-density lipoprotein cholesterol levels, and BMI between high-intensity interval and moderate-intensity continuous training groups [40]. Although the same exercise training modalities (supervised AET, supervised RT, and AET + RT) were included in a study by Pan et al. [41], their subjects had type 2 diabetes. They indicated that AET + RT intervention showed more pronounced improvement in HbA1c levels and a less significant improvement in some of the cardiovascular risk factors compared to the other interventions [41]. In addition, Radhika et al. [42] suggested that physical activity interventions improved oral glucose tolerance, FBG and HbA1C levels, VO_2_max, and body composition slightly more in prediabetic patients than in the control group but without a significant difference. However, the types of physical activity interventions were not studied further. These results were partially confirmed by the present study’s findings showing that physical activity interventions, either AET or RT alone, could significantly reduce HbA1C levels in prediabetic patients.

This study was the first to compare the effects of four exercise training interventions (AET + RT, AET, RT, and CT) in prediabetic patients. However, several limitations of this study should be mentioned. A major limitation of the present meta-analysis is that the overwhelmingly lower number of studies on evaluating AET + RT and RT interventions compared to AET intervention may have influenced the pooled results. In addition, the intensity of RT is determinative of its benefits but has not been specifically described in the majority of the studies on RT. These two key limitations may have led to a possible underestimation of the curative effects of RT or AET + RT when compared to AET. Therefore, it is important to focus on studies on AET + RT and RT interventions with a specific emphasis on the intensity of these training intervention programs. Other limitations include the following: First, significant heterogeneity was found, probably owing to the different subjects with IFG or IGT and different study regions, which could have acted as potential confounders that influenced the results of the meta-analysis. Second, a majority of the included studies originated in Europe and the Americas, which could have led to a selection bias. Third, ranking of the P-scores using fixed- and random-effects models was not consistent. Fourth, the overall quality of the present study was moderate. All the included studies demonstrated a high risk of performance bias. Fifth, the response of blood glucose levels and insulin secretion to insulin resistance varies with exercise in an intensity-dependent manner [32]. Different versions of the four exercise programs (AET, RT, AET + RT, or CT) were performed in the included studies, and even in studies of the same exercise intervention, the exercise intensity differed. Comparisons of exercises with specific intensities were limited by future subgroup analyses, resulting in an intensity bias in the findings of this study. Sixth, most comparisons for indicators were reported in only one study, limiting the reliability and validity of the findings. Finally, the P-score used in this study was mainly based on the effect size of the intervention trial, and does not reflect the influence of accuracy (the size of the confidence interval) on the results. Furthermore, more high-quality RCTs conducting multiple comparisons among different indicators, including safety, optimal exercise intensity, and duration, are needed for future investigations.

## Conclusions

AET, AET + RT, and RT interventions exerted beneficial effects on patients with prediabetes. AET or AET + RT interventions were superior for partial improvement in BMI values, insulin levels, HOMA-IR, FBG, and HbA1c levels compared with RT, although the lower number of subjects on RT and the lower quality of assessment of methods may have underestimated RT’s beneficial effects when compared to the other modalities. Further investigations to validate these findings are required because studies focusing on the specific effects of AET + RT and RT interventions on prediabetes and glucose-related parameters are still lacking. It is recommended that RCTs with the capacity for multiple comparisons using indicators focused on safety, optimal exercise intensity, and duration are conducted to better understand the efficacy of these interventions.

## Supplementary Information

**Additional file 1: Fig. S1** Forest plot of standard mean difference to compare body mass index values in prediabetic patients treated with different exercise interventions. Squares indicate the estimates for the corresponding study, and the size of the square is proportional to the weight of the study to the overall estimate. Diamonds indicate the overall pooled estimate, and the horizontal lines represent the 95% confidence interval.

**Additional file 2: Fig. S2** Forest plot of standard mean difference to compare fasting blood glucose levels in prediabetic patients treated with different exercise interventions.

**Additional file 3: Fig. S3** Forest plot of standard mean difference to compare HbA1c levels in prediabetic patients treated with different exercise interventions.

**Additional file 4: Fig. S4** Forest plot of standard mean difference to compare insulin levels in prediabetic patients treated with different exercise interventions.

**Additional file 5: Fig. S5** Forest plot of standard mean difference to compare the homeostatic model assessment of insulin resistance index values in prediabetic patients treated with different exercise interventions.

**Additional file 6: Supplementary Table 1** Retrieval steps and results of PubMed search (retrieval time: 2020911).

**Additional file 7: Supplementary Table 2** Retrieval steps and results of Embase search.

**Additional file 8: Supplementary Table 3** Retrieval steps and results of the Cochrane Library search.

**Additional file 9: Supplementary Table 4** Retrieval steps and results of the SPORTDiscus search.

## Data Availability

Data sharing is not applicable to this article as no new data were created or analyzed in this study.

## Abbreviations

AET: Aerobic exercise training
BMI: Body mass index
CT: Control training
CI: Confidence interval
FBG: Fasting blood glucose
HbA1c: Glycated hemoglobin
HOMA-IR: Homeostasis model assessment of insulin resistance index
IFG: Impaired fasting glucose
IGR: Impaired glucose regulation
IGT: Impaired glucose tolerance
RT: Resistance training
SMD: Standardized mean difference
RCT: Randomized controlled study
VO_2_max: Maximal oxygen consumption
