# Supplementary figures and images for: Comparisons of different exercise interventions on glycemic control and insulin resistance in prediabetes: a network meta-analysis
Source: BMC Endocr Disord. 2021 Sep 6;21:181. doi: 10.1186/s12902-021-00846-y (PMC8422751; doi:10.1186/s12902-021-00846-y)

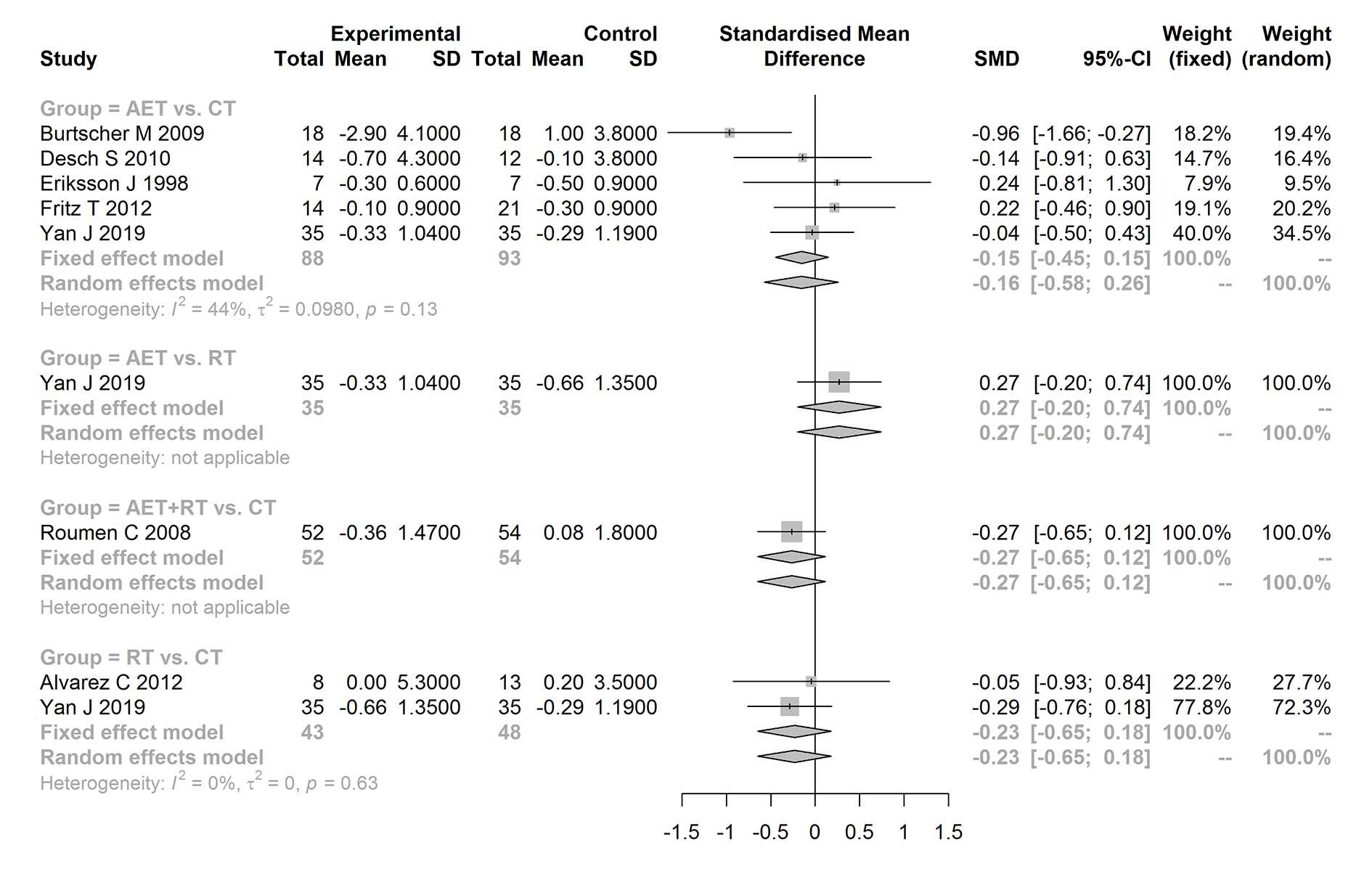

Supplement: Supplementary file 1 — Additional file 1: Fig. S1 Forest plot of standard mean difference to compare body mass index values in prediabetic patients treated with different exercise interventions. Squares indicate the estimates for the corresponding study, and the size of the square is proportional to the weight of the study to the overall estimate. Diamonds indicate the overall pooled estimate, and the horizontal lines represent the 95% confidence interval. [file 12902_2021_846_MOESM1_ESM.tif]

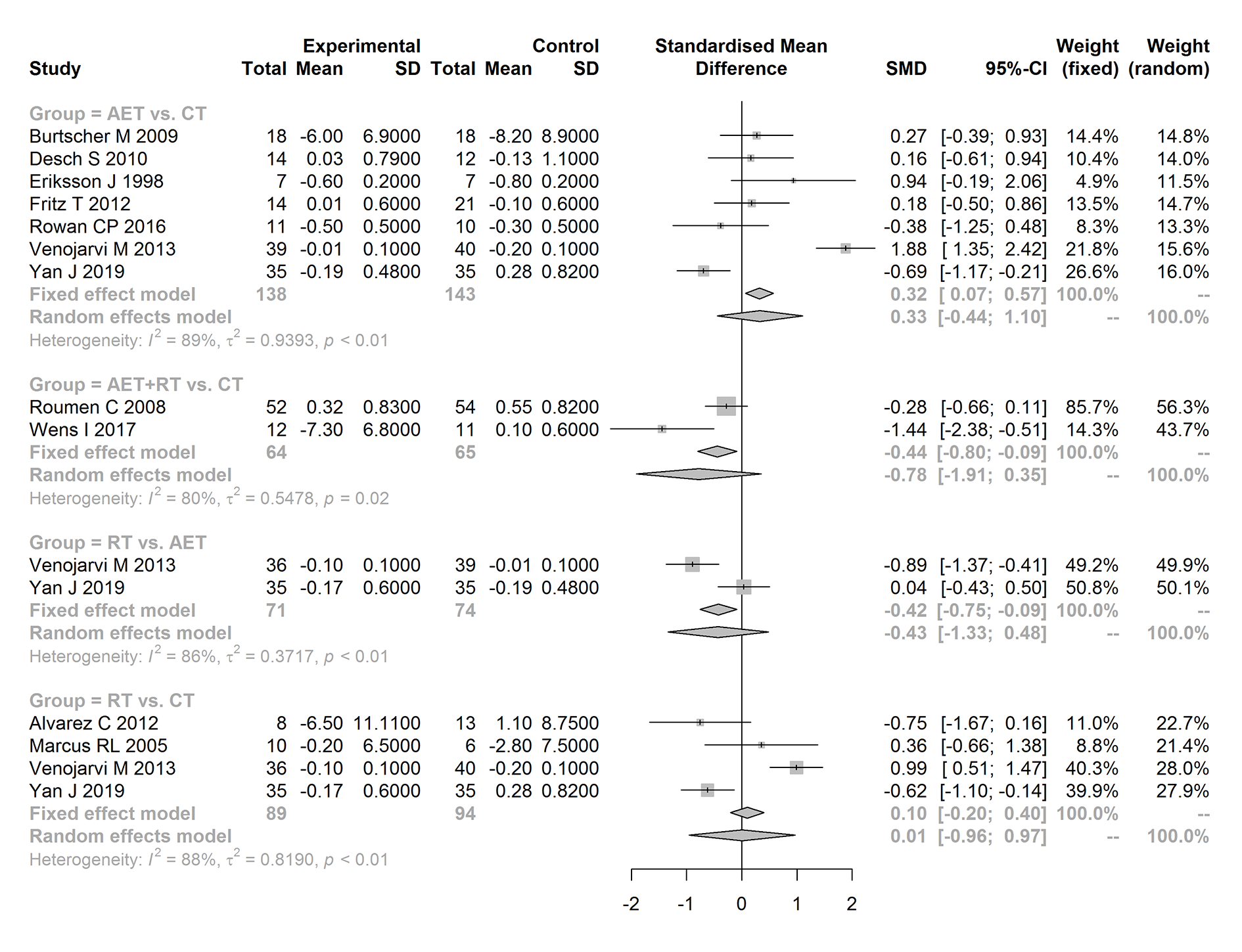

Supplement: Supplementary file 2 — Additional file 2: Fig. S2 Forest plot of standard mean difference to compare fasting blood glucose levels in prediabetic patients treated with different exercise interventions. [file 12902_2021_846_MOESM2_ESM.tif]

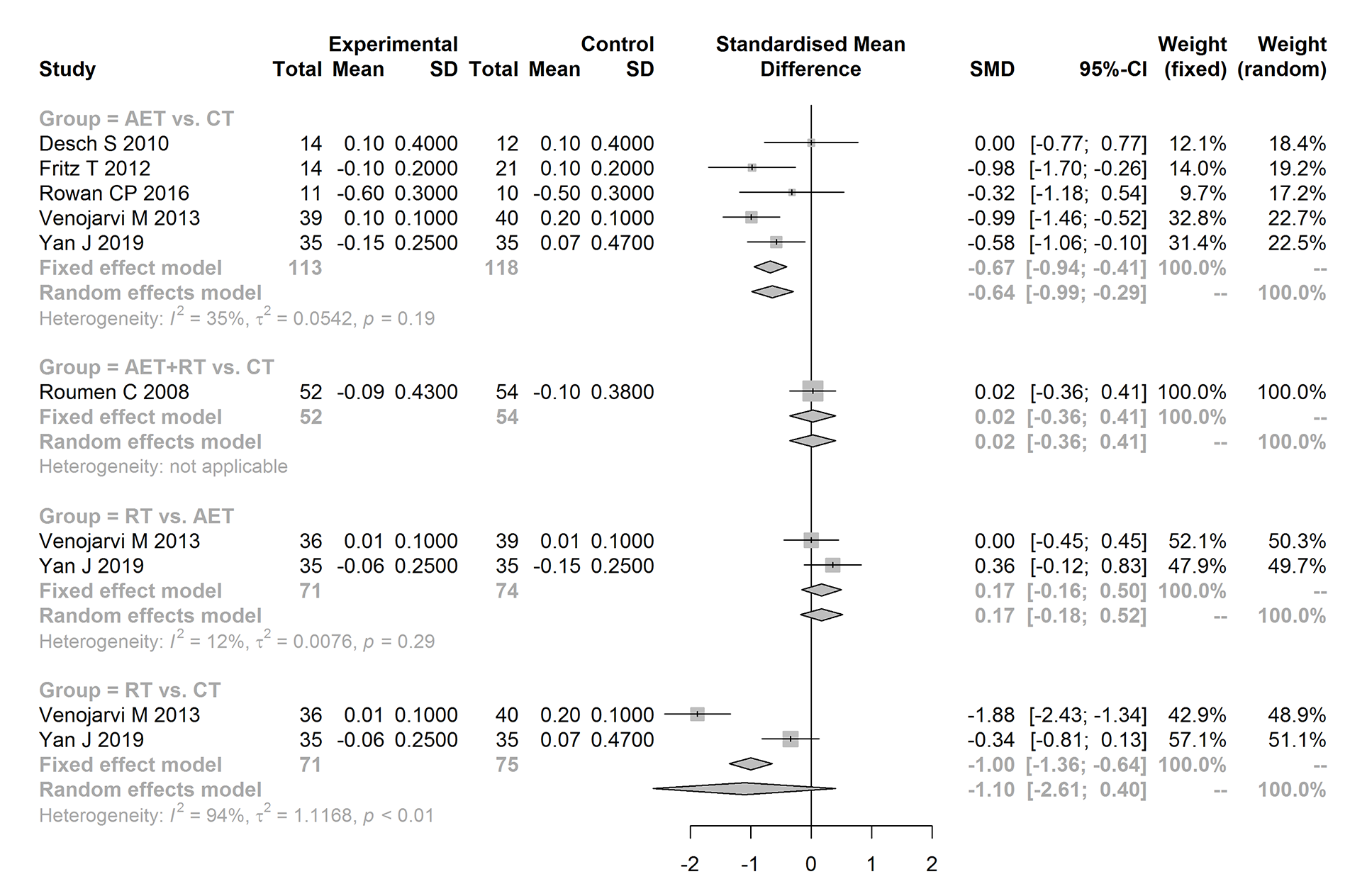

Supplement: Supplementary file 3 — Additional file 3: Fig. S3 Forest plot of standard mean difference to compare HbA1c levels in prediabetic patients treated with different exercise interventions. [file 12902_2021_846_MOESM3_ESM.tif]

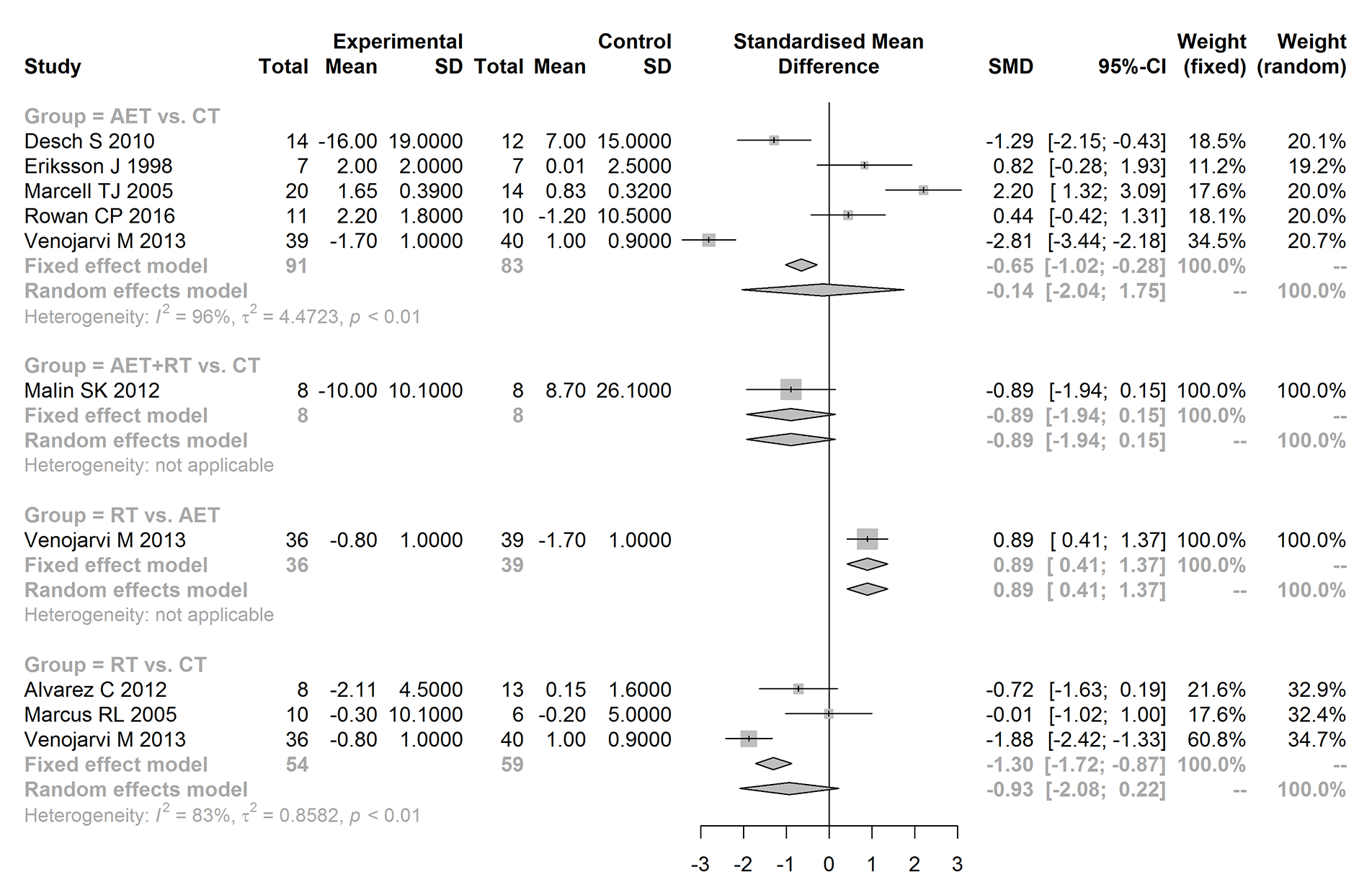

Supplement: Supplementary file 4 — Additional file 4: Fig. S4 Forest plot of standard mean difference to compare insulin levels in prediabetic patients treated with different exercise interventions. [file 12902_2021_846_MOESM4_ESM.tif]

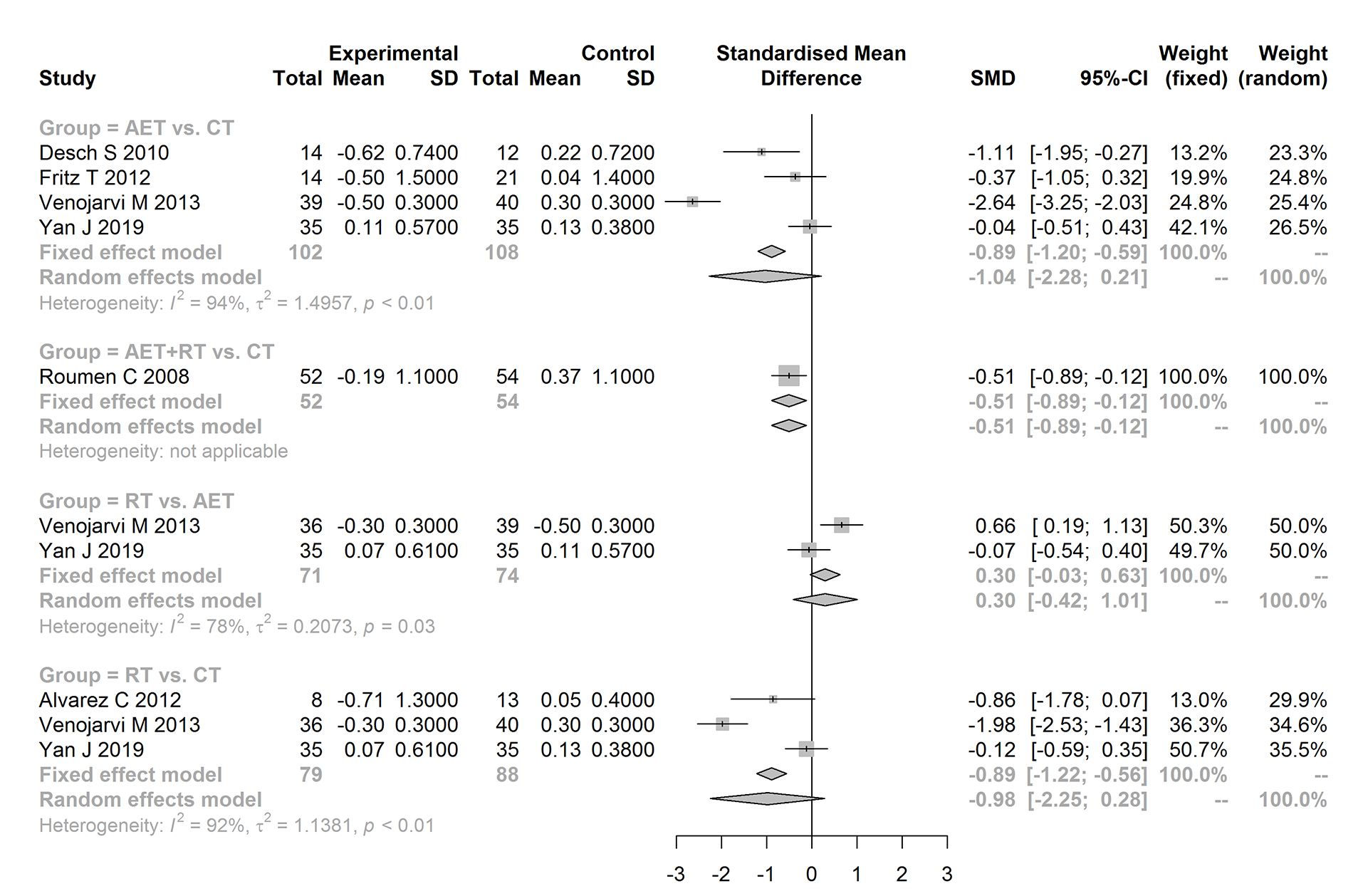

Supplement: Supplementary file 5 — Additional file 5: Fig. S5 Forest plot of standard mean difference to compare the homeostatic model assessment of insulin resistance index values in prediabetic patients treated with different exercise interventions. [file 12902_2021_846_MOESM5_ESM.tif]
