# Supplementary material for: Comparisons of different exercise interventions on glycemic control and insulin resistance in prediabetes: a network meta-analysis
Source: BMC Endocr Disord. 2021 Sep 6;21:181. doi: 10.1186/s12902-021-00846-y (PMC8422751; doi:10.1186/s12902-021-00846-y)
Supplement: Supplementary file 6 — Additional file 6: Supplementary Table 1 Retrieval steps and results of PubMed search (retrieval time: 2020911). [file 12902_2021_846_MOESM6_ESM.docx]

**Supplementary Table 1 Retrieval steps and results of PubMed search (retrieval time: 2020911)**

| Search | Query | Items found |
| --- | --- | --- |
| #1 | "exercise"[MeSH Terms] OR "exercise"[All Fields] OR "exercises"[All Fields] OR "exercise therapy"[MeSH Terms] OR "exercise therapy"[All Fields] OR "exercised"[All Fields] OR "exerciser"[All Fields] OR "exercisers"[All Fields] OR "exercising"[All Fields] OR ("weightlifter"[All Fields] OR "weightlifters"[All Fields] OR "weightlifting"[All Fields]) OR "aerobic exercise"[All Fields] OR "aerobic training"[All Fields] OR ("movement"[MeSH Terms] OR "movement"[All Fields] OR "movements"[All Fields] OR "movement s"[All Fields]) OR "physical therapy"[All Fields] OR "resistance exercise"[All Fields] OR "physical activity"[All Fields] OR "resistance training"[All Fields] OR "resistance therapy"[All Fields] | 1,200,950 |
| #2 | "prediabetic state"[MeSH Terms] OR ("prediabetic"[All Fields] AND "state"[All Fields]) OR "prediabetic state"[All Fields] OR ("pre"[All Fields] AND "diabetes"[All Fields]) OR "pre diabetes"[All Fields] OR ("prediabetes"[All Fields] OR "prediabetic"[All Fields] OR "prediabetics"[All Fields]) OR "impaired glucose regulation"[All Fields] OR "IGR"[All Fields] OR "impaired fasting glucose"[All Fields] OR "IFG"[All Fields] OR "impaired glucose tolerance"[All Fields] OR "IGT"[All Fields] OR "glucose metabolism disorders"[All Fields] OR "glucose alterations"[All Fields] OR "dysglycemia"[All Fields] OR"hyperglycemia"[All Fields] OR "dysglycemias"[All Fields] | 50,669 |
| #3 | #1 AND #2 | 4621 |
| #4 | #3 AND (((randomized controlled trial[pt]) OR (controlled clinical trial[pt]) OR (randomized[tiab] OR randomised[tiab]) OR (placebo[tiab]) OR (drug therapy[sh]) OR (randomly[tiab]) OR (trial[tiab]) OR (groups[tiab])) NOT (animals[mh] NOT humans[mh])) | 1944 |
