# Supplementary material for: Comparisons of different exercise interventions on glycemic control and insulin resistance in prediabetes: a network meta-analysis
Source: BMC Endocr Disord. 2021 Sep 6;21:181. doi: 10.1186/s12902-021-00846-y (PMC8422751; doi:10.1186/s12902-021-00846-y)
Supplement: Supplementary file 7 — Additional file 7: Supplementary Table 2 Retrieval steps and results of Embase search. [file 12902_2021_846_MOESM7_ESM.docx]

**Supplementary Table 2 Retrieval steps and results of Embase search**

| Search | Query | Items found |
| --- | --- | --- |
| #1 | 'exercise'/exp OR exercise OR weightlifting OR 'aerobic exercise'/exp OR 'aerobic exercise' OR 'aerobic training'/exp OR 'aerobic training' OR 'aerobic therapy' OR 'movement'/exp OR movement OR 'physical therapy'/exp OR 'physical therapy' OR 'resistance exercise'/exp OR 'resistance exercise' OR 'physical activity'/exp OR 'physical activity' OR 'resistance training'/exp OR 'resistance training' OR 'resistance therapy' | 1,540,961 |
| #2 | 'prediabetes' OR prediabetic OR 'impaired glucose regulation'/exp OR 'impaired glucose regulation' OR IGR OR 'impaired fasting glucose'/exp OR 'impaired fasting glucose' OR IFG OR 'impaired glucose tolerance'/exp OR 'impaired glucose tolerance' OR IGT OR 'glucose metabolism disorders' OR 'glucose alterations' OR 'hyperglycemia 'OR 'dysglycemia'/exp OR dysglycemia | 57,892 |
| #3 | #1 AND #2 | 6508 |
| #4 | #3 AND (crossover AND ('procedure'/exp OR procedure) OR (double AND ('blind'/exp OR blind) AND ('procedure'/exp OR procedure)) OR (randomized AND controlled AND ('trial'/exp OR trial)) OR ('single blind' AND ('procedure'/exp OR procedure)) OR random* OR factorial* OR crossover* OR (cross AND over*) OR placebo* OR (doubl* AND adj AND blind*) OR (singl* AND adj AND blind*) OR assign* OR allocat* OR volunteer*) | 2069 |
