# Supplementary material for: Comparisons of different exercise interventions on glycemic control and insulin resistance in prediabetes: a network meta-analysis
Source: BMC Endocr Disord. 2021 Sep 6;21:181. doi: 10.1186/s12902-021-00846-y (PMC8422751; doi:10.1186/s12902-021-00846-y)
Supplement: Supplementary file 8 — Additional file 8: Supplementary Table 3 Retrieval steps and results of the Cochrane Library search. [file 12902_2021_846_MOESM8_ESM.docx]

**Supplementary Table 3 Retrieval steps and results of the Cochrane Library search**

| Search | Query | Items found |
| --- | --- | --- |
| #1 | MeSH descriptor: [Exercise] explode all trees | 24,066 |
| #2 | ((exercise OR weightlifting OR “aerobic exercise” OR “aerobic training” OR “aerobic therapy” OR movement OR “physical therapy” OR “resistance exercise” OR “physical activity” OR “resistance training” OR “resistance therapy”)):ti,ab,kw | 147,632 |
| #3 | #1 OR #2 | 149,300 |
| #4 | MeSH descriptor: [Prediabetic State] explode all trees | 975 |
| #5 | ((pre-diabetes OR prediabetic OR “impaired glucose regulation” OR “impaired fasting glucose” OR “impaired glucose tolerance” OR “glucose metabolism disorders” OR “glucose alterations” OR “hyperglycemia” OR dysglycemia)):ti,ab,kw | 4966 |
| #6 | #4 OR #5 | 4966 |
| #7 | #3 AND #6 | 1294 |
| #8 | #7 in trials | 1287 |
