# Supplementary material for: Comparisons of different exercise interventions on glycemic control and insulin resistance in prediabetes: a network meta-analysis
Source: BMC Endocr Disord. 2021 Sep 6;21:181. doi: 10.1186/s12902-021-00846-y (PMC8422751; doi:10.1186/s12902-021-00846-y)
Supplement: Supplementary file 9 — Additional file 9: Supplementary Table 4 Retrieval steps and results of the SPORTDiscus search. [file 12902_2021_846_MOESM9_ESM.docx]

**Supplementary Table 4 Retrieval steps and results of the SPORTDiscus search**

| Search | Query | Items found |
| --- | --- | --- |
| #1 | TX (exercise OR weightlifting OR “aerobic exercise” OR “aerobic training” OR “aerobic therapy” OR movement OR “physical therapy” OR “resistance exercise” OR “physical activity” OR “resistance training” OR “resistance therapy”) | 415,981 |
| #2 | TX (pre-diabetes OR prediabetic OR “impaired glucose regulation” OR IGR OR “impaired fasting glucose” OR IFG OR “impaired glucose tolerance” OR IGT OR “glucose metabolism disorders” OR “glucose alterations” OR “hyperglycemia” OR “dysglycemia”) | 2773 |
| #3 | TX (((“randomized controlled trial”) OR (“controlled clinical trial”) OR randomized OR randomised OR placebo OR randomly OR trial)) | 182,211 |
| #4 | #1 AND #2 AND #3 | 1149 |
